# The British Medical Association

**Published:** 1901-07-27

**Authors:** 


### The Hospital

A Journal of

The Medical Sciences and Hospital Administration.

Vol. XXX.—No. 774.] Edited by Sir Henry Burdett, K.C.B., and Solomon C. Smith, M.D., M.R.C.P. [July 27, 1901.

### The British Medical Association.

From the programme which has been published it is evident that the annual meeting of the British Medical Association, which will be held next week at Cheltenham, will not be behind its predecessors in professional and social interest. There are circumstances, however, which render this a specially momentous occasion in the history of the Association, for the report of the constitution committee, which was appointed last year, will have to be considered, and upon the reception given to it will depend in large degree the future of the Association. In broad outline the effect of the proposed new constitution will be on the one hand to limit considerably the independence of the Council, for although the executive power will, as hitherto, remain with the Council, this body will in future have to carry out the policy of the members as expressed by the meeting of representatives.

On the other hand, the duties of the general meetings will be strictly limited to the election of officers and the passing of the accounts, and all proposals affecting the by-laws, the disposal of the property of the Association, or the honour and interests of the medical profession, instead of being discussed by Dick, Tom, and Harry, at irresponsible general meetings, will come before the representatives sent up from the various localities (Divisions as they are called), and these gentlemen will have to give their votes as they are directed by their constituents—if their constituents take the trouble to make up their minds on the subject.

All resolutions passed by this meeting of representatives will be regarded as the decision of the Association and will be binding on the Council—with this reservation, however, that in any case where the Council is of opinion that such resolution does not properly represent the wishes of the Association, the Council will be able, within four months, to put in force the referendum, and to refer the matter to special meetings of all the Divisions. Thus is it hoped to clip the wings of the bores and orators, and at the same time to put an end to the scandal of a Council year after year refusing to act upon the definite instructions of a general meeting. On the whole the new constitution seems a good and rather clever bit of machinery for enabling all and sundry to exercise some little sway in the conduct of the Association, while at the same time preventing its funds and policy falling under the control of a few masterful individuals. Whether the members at large are possessed of any great desire to exercise this influence in the councils of the Association is another matter, as to which we have our doubts. But ingenious as the scheme may be, it is expensive: much money goes in the payment of the representatives, and we fancy many people will be found to question whether it is wise even for so rich a body as the British Medical Association to spend so much upon the mere machinery of self-government, a matter as to which the vast majority of its members have shown themselves absolutely and hopelessly indifferent.

There are some, however, to whom these matters are not indifferent, and one cloud which just now hangs over the Association is the position of its colonial Branches. Some of these are at present in a very independent humour, and the recent federation of the Australian governments is hardly likely to make the Branches in that country less urgent in their demand for autonomy. The trades-union sentiment there is very strong, and they are very anxious to get into their own hands the power of expelling their members from the Association—a gentle whip which they will no doubt apply *con amore* for the flagellation of their recalcitrant members. Which is all very well, but is hardly to the taste of the parent Association, which has already been dragged in the mire quite enough by their proceedings. The question, however, is to be referred to a committee, which is rather like asking the Association to bury its head in the sand. There will be trouble with these distant colonial Branches, whose sole link to the Association is its journal. Indeed there will be trouble all round if the power of expulsion should be prostituted to political and trades union purposes; and, unfortunately, a strong desire is evident among some of the members to give but a short shrift to those who decline to bow meekly to the will of the majority. For example, among the various matters to be discussed at Cheltenham is a motion, of which notice has been given, to the effect that no member of a Branch shall oppose or enter into competition with any other member in any course of action which he may take in connection with any public appointment which he may hold, so long as such a course of action is sanctioned by, or arises out of advice given by, the

Council of the Branch. This applies to efforts to obtain a higher rate of remuneration, or to obtain an increase of salary. It is, of course, trades unionism pure and simple; and any infraction of this rule is to be punished by expulsion from the Branch! We do not say that there is no opening for a trades union among doctors. Probably there is; and if there is, it only wants an energetic secretary to set the ball a-rolling; but to attempt by a mere passing of a by-law to turn a respectable semi-scientific Association into a trades union is somewhat too barefaced a proceeding. It probably will come to nothing; still it serves to show which way the wind blows.

